# Self-Construal as a Mediator Between Identity Structure and Subjective Well-Being

**DOI:** 10.1007/s12144-013-9202-5

**Published:** 2014-01-24

**Authors:** Aleksandra Pilarska

**Affiliations:** Department of Personality Psychology, Institute of Psychology, Adam Mickiewicz University, Szamarzewskiego 89, 60-568 Poznań, Poland

**Keywords:** Independent self-construal, Interdependent self-construal, Identity structure, Subjective well-being

## Abstract

An examination of the assumptions underlying identity conceptualizations in psychology of self indicates the assumptions are based on an independent, individualistic view of self. If self is constructed as interdependent with others, such identity characteristic as a sense of uniqueness, separateness, and continuity may be less important in promoting well-being. The results of the conducted study (*N* = 226) indicated that there were weaker relations between various features of identity structure and subjective well-being for individuals with a highly interdependent self-construal than for those with a highly independent self-construal. The results also showed that specificity, separateness, and stability of identity content influenced positive and negative affect through the mediating agency of independent and interdependent self-construals. These findings emphasize the importance of applying a self-construal perspective in considering adaptive functions of identity.

## Introduction

Most of the traditional conceptualizations of identity indicate that the process of development in its normative course leads to the formation of a consolidated identity based on an individual’s (mostly internal) attributes that are of fundamental importance for him or her (e.g., Erikson 1963, 1968; Schachter 2002). Forming a separate, coherent, unique, and stable identity is regarded as critical for further development, being a kind of “landmark in psychosocial maturity of the human being” (Straś-Romanowska 2008, p. 21). Loss of separateness, specificity, continuity, and coherence of identity is considered as having a definitely negative impact on an individual’s emotional well-being (e.g., Chen et al. 2007; Erikson 1980; Grzegolowska-Klarkowska and Zolnierczyk 1988; Schwartz et al. 2010; Vignoles et al. 2000). This kind of description of identity is part of a broader tendency to regard the pursuit of consistency, individuality, separateness from social context, and self-enhancement, as both essential manifestations of self and significant indicators of effective adaptation and mental health (e.g., Block 1961; Donahue et al. 1993; Jarymowicz and Codol 1979; Lecky 1945 as cited in Swann 1990; Rogers 1959; Sedikides 1993; Swann et al. 2005).

In the last decades there has been an increased interest in the field of self psychology. A number of important theoretical distinctions have been introduced regarding the different facets of self (e.g., Greenwald and Pratkanis 1988; Hermans 2003; Higgins 1987; Markus and Kunda 1986; McAdams 2013a; Triandis 1989; Turner and Reynolds 2012). Among these new approaches to the self, one of the most promising and widely used is the self-construal approach (Markus and Kitayama 1991). The basic idea of this paradigm is that there are two different types of self-concept, interdependent and independent. The notion of self-construal suggests that the majority of previous approaches to identity were predominantly based on the model of the private self, separated from social roles and relations, and defined through dispositions, qualities, capabilities, and goals (Markus and Kitayama 1991). This independent view of the self, however, fails to describe the self-views of all people. An individual may define oneself in terms of significant, close relationships, and in this way construe his or her self as interdependent. The new perspective questions the universal relevance and nature of a wide range of psychological processes, including those related to identity formation and self-experience. This suggests a necessity to review at least some of the findings in this field and calls for a more refined model of identity development and its adaptive functions.

This article is intended to provide an empirically based rationale for including the two types of self-construal as important variables in understanding the linkages between identity and subjective well-being.

### Independent and Interdependent Self-Construal

The concept of self-construal seems to be rooted in at least three traditions. On the one hand, it attempts to link the cultural dimension of individualism–collectivism with personality; secondly, it is a part of stormy discussions of the past decades on the complexity of self. And finally, it reflects the basic, though conflicting, human drives for both individuation and affiliation (e.g., Benjamin 1974, 1996; Bowlby 1969; Franz and White 1985; Hermans 1999; Imamoğlu 2003; Maslow 1970). In the broadest sense, the term *self-construal* refers to the way an individual understands oneself in relation to other people, and, arising out of this self-understanding, the view of self as essentially independent and separate from others, or, on the other hand, interdependent with others and never fully differentiated from the social context. Self-construal is therefore conceptualized as a “constellation of thoughts, feelings, and actions concerning one’s relationship to others, and self as distinct from others” (Singelis 1994, p. 581). The theoretical perspective adopted in this paper emphasizes the role of a fundamental tension (or opposition) between motives and goals that promote individuation, and those that promote affiliation in generating individual differences in self-construals (Guisinger and Blatt 1994; Kagitcibasi 2005; Markus and Kitayama 1991; Singelis 1994). Others have developed formulations that refer to differences in the perspective people take vis-à-vis the self (Kitayama and Imada 2008), different variants of propositional and, to a lesser extent, conceptual self-representations (Schlicht et al. 2009), different levels of inclusiveness of the conceptualization of the self (Brewer and Gardner 1996), and different degrees and extents to which individuals are linked in a social network (Muthuswamy 2007). Also, a number of writers have further elaborated on the nature of interdependent self-construal and distinguished different facets of interdependence, namely harmony seeking and rejection avoidance (Hashimoto and Yamagishi 2013), and relational and collective interdependence (Gabriel and Gardner 1999; Kashima and Hardie 2000). Although these approaches focus on different aspects of self-construals, they need not be considered as antithetical or contradictory.

Since both individuation and affiliation comprise universal human motives, each individual may have both independent and interdependent self-construals. The relative strength and accessibility of these self-construals on a chronic basis are, however, specific to the individual and reflect the individual’s balance between individuation and affiliation (Brewer and Gardner 1996; Imamoğlu 2003; Singelis 1994). Such a balance is affected to a great extent by the particular socio-cultural and familial context that a person is embedded within (Adams 1998; Kagitcibasi 2005). And so an individual’s predominant self-construal is largely determined by his or her interpersonal experiences and socio-cultural milieu (e.g., Imamoğlu 2003; Markus and Kitayama 1991, 2003; Triandis 1989; Triandis and Suh 2002). It is worthwhile to mention that, regardless of whether one’s dominant self-construal is independent or interdependent, both self-construals may be shaped by temporary contextual influences as well. In fact, all individuals may be expected to flexibly define themselves as relatively more independent or interdependent depending on current motives or the current situation (e.g., Gardner et al. 1999; Trafimow et al. 1991). Self-construal, therefore, should be described in relative rather than absolute terms.

### Identity Structure

In the field of psychology, identity is most frequently defined by reference to (a) subjective self-experience (e.g., Blasi and Glodis 1995; Sokolik 1996; Vignoles et al. 2006), (b) cognitive structure (e.g., Berzonsky 2004; Jarymowicz 1989; Kozielecki 1986), (c) axiological orientation (e.g., Berzonsky 2004; Freud 1956 as cited in Erikson 1980), and (d) life history (e.g., McAdams 2013b). Although the views of various authors on what identity actually means vary, most of them agree that it is the individual’s self that provides the basis for identity (e.g., Erikson 1980; James 1890; Grzelak and Jarymowicz 2002). It is important to note here that the distinction between self and identity is not consistently well-established and the two concepts are sometimes used interchangeably in the literature (e.g., Baumeister and Muraven 1996; Swann and Bosson 2010). Nevertheless, many authors argue that the distinction should be made and maintained (e.g., Berzonsky 2005; Katzko 2003; Oleś 2008). From the research findings on both self and identity, it is not only possible, but also worthwhile to distinguish between self (or self-concept) and identity. This can be done in terms of content, organization, and functions. The self can be regarded as a broader and superordinate concept, and identity may be considered as an expression of self, a particular sub-component, or a specific aspect of the self—in other words, a kind of “extract” from the self (e.g., Erikson 1980; James 1890; Oleś 2008; Owens 2006; Pilarska and Suchańska 2013).^1^ Identity, in this view, comprises those self-representations which are key for defining oneself and differentiating the self from the non-self. Furthermore, there is an agreement that the self is a multifaceted phenomenon consisting of a number of self-conceptions which change depending on the situational context, role, and relations with others (e.g., Greenwald and Pratkanis 1988; Markus and Wurf 1987; Roberts 2007; Suszek 2007; Swann and Bosson 2010). Over the past decades, this notion of a multifaceted self (self-pluralism, poly-psychism, multiphrenia, etc.) has proven so appealing that it seems difficult to provide a cohesive model of the multiple self (see McConnell 2011; Rowan and Cooper 1999; Suszek 2007; Swann and Bosson 2010). However, the same cannot be said about identity, since, by definition, it implies singularity, sameness, and continuity. We may therefore think in terms of multiple identifications or various elements (dimensions or domains) of identity, but the concept of *multiple identities* puts together mutually exclusive terms (e.g., Berzonsky 2005; Erikson 1968; Kroger 2004, 2005). Thus, the conception of a unitary identity that brings about (or fails to achieve) integration within the self is favored here.

For the purpose of this research, personal identity is defined as a psychological structure that organizes the individual’s self experience (see also Berzonsky 2004; Kroger 2005). Each individual’s identity may be based on various elements through which an individual defines oneself, such as competencies, values, commitments, and roles, and which make up the content of identity. In other words, an individual’s identity consists of those self-characteristics that an individual considers most representative of oneself and most important, so that their loss would evoke a feeling that one is no longer the same person (see also Kozielecki 1986; Markus 1977; Oleś 2008). In the first person (phenomenological) perspective, identity corresponds to the recurring mode of experiencing oneself-as-subject. Within this understanding of identity, the structural features of identity manifest themselves in a variety of identity-related senses. These senses, according to the approach presented here, can be described by the following dimensions:

- Accessibility of identity content: The clarity and ease in retrieving the content of identity, referred to as a sense of having inner contents (thoughts, feelings, etc.).
- Specificity of identity content: The distinctiveness and uniqueness of the content of identity, referred to as a sense of uniqueness.
- Separateness of identity content: The constancy in differentiating the content of identity (in terms of self versus non-self), referred to as a sense of one’s own boundaries.
- Coherence of identity content: The internal consistency and congruence of the content of identity, referred to as a sense of coherence.
- Stability of identity content: The temporal stability of the content of identity, referred to as a sense of continuity over time.
- Valuation of identity content: The positivity of the content of identity, referred to as a sense of self-worth.

The above structural dimensions of identity are built on three main sources. The first is clinical observations of psychotic patients having identity disorders (e.g., Rothschild 2009; Sokolik 1996); the second derives from Erikson’s (1968, 1980) and his followers’ (e.g., Mallory 1989; Marcia 1966) works; the third is grounded in the analysis of semantic content of terms used to describe the structure of identity in psychological literature. Table 1 lists many of these terms.

**Table 1:** Identity dimensions and the corresponding terms

Identity dimension	Corresponding terms	References
Accessibility	Inner emptiness	e.g., Soloman 2004; Winston 2005
Specificity	Self-uniqueness, self-distinctiveness (distinctness)	e.g., Baumeister and Muraven 1996; Blasi 1993; Breakwell 1993; Damon and Hart 1988; Maslow 1970; Oleś 2008; Straś-Romanowska 2008; Vignoles et al. 2006
Separateness	Individuality, self-other boundaries	e.g., Blasi 1993; Blatt and Levy 2003; Guidano 1987; Köpke 2012; Schachter 2005; Sollberger 2013
Coherence	Inner integration and homogeneity, inner coherence, self-unity, identity discrepancy, identity consistency, self-clarity	e.g., Berzonsky 2005; Blasi 1993; Blasi and Glodis 1995; Campbell 1990; Campbell et al. 2003; Dhar et al. 2010; Giddens 1991; Goth et al. 2012; Greenwald and Pratkanis 1988; Killeya-Jones 2005; Mandrosz-Wróblewska 1989; Maslow 1970; Schachter 2005; Sollberger 2013; Straś-Romanowska 2008; Suh 2002
Stability	Self-continuity, (internal) stability, self-sameness, self-clarity	e.g., Breakwell 1993; Campbell 1990; Damon and Hart 1988; Giddens 1991; Goth et al. 2012; Greenwald and Pratkanis 1988; Maslow 1970; Oleś 2008; Schachter 2005; Sollberger 2013; Straś-Romanowska 2008; Vignoles et al. 2006
Valuation	Self-worth, self-efficacy, self-regard	e.g., Breakwell 1993; Greenwald and Pratkanis 1988; Oleś 2008; Rogers 1959; Vignoles et al. 2006

### Self-Construal and the Features of Identity Structure

Identity is a dynamic phenomenon—both the content and the structure of identity may change over time due to multiple motives, such as self-worth, continuity, belonging, distinctiveness, and coherence (e.g., Breakwell 2010; Oleś 2008; Vignoles et al. 2006). The relative strength of any of these motives varies between and within individuals and determines the features of an individual’s identity. The interplay between self and identity allows the assumption that the way the self is construed will contribute to these differences. An independent or interdependent self-construal shapes the axiological and motivational constituents that go into forming and maintaining identity.^2^

Construing one’s self as an autonomous individual implies a desire to discover, understand and express one’s personal attributes, as well as to confirm one’s uniqueness and separateness from others (Markus and Oyserman 1989; Markus and Kitayama 1991; Singelis 1994). Characteristic for people with an independent self-construal are: greater differentiation of self-representation from the representation of others (Kühnen and Hannover 2000; Madson and Trafimow 2001); describing oneself as having distinctive characteristics that are not shared by others (Cross et al. 2003; Stapel and Koomen 2001; Triandis 1989); a tendency to underestimate one’s similarity to others (Markus and Kitayama 1991); a context-independent processing style (Kühnen et al. 2001); a propensity to take actions aimed at differentiating oneself from others, as evidenced by a diminished tendency to mimicking behavior (van Baaren et al. 2003). Given the above, people with an independent self are more likely to report a strong sense of uniqueness and boundaries.

On the other hand, construing one’s self as interdependent and embedded in social relationships implies a strong orientation to relational and other environmental contexts. The overriding goal in this case is to maintain a sense of belongingness and harmonious fit with others (Markus and Oyserman 1989; Markus and Kitayama 1991). Characteristic of people with an interdependent self-construal are: describing oneself in terms of attributes that are shared with others (Locke and Christensen 2007; McGuire and McGuire 1988 as cited in Cross et al. 2002; Triandis 1989); an increased perceived self-other similarity and a tendency to overestimate one’s similarity to others (Cross et al. 2002; Kühnen and Hannover 2000; Markus and Kitayama 1991); an integration mind-set and inclusion of others in the self (Downie et al. 2006; Kashima and Hardie 2000; Stapel and Koomen 2001); taking into account others’ needs and wishes when making decisions and reliance on others’ beliefs as reasons for one’s judgment (Cross et al. 2000; Torelli 2006). Therefore, those with an interdependent self-construal are likely to be less inclined to strive for a sense of uniqueness and separateness.

The way in which one construes the self has an impact also on one’s identity coherence and stability. Independent self-construal entails the belief that there is a core authentic self, one “real nature” of an individual (Cross et al. 2003). A stable and consistent expression of traits, abilities, attitudes, and other characteristics underlies the definition and validation of this real self. Inconsistency and a loss of a sense of continuity pose a threat to the core self and may lead to confusion within the self-concept, a lack of self-clarity, and experiencing the self as fragmented (Campbell 1990; Church et al. 2008; Cross et al. 2003; Donahue et al. 1993).

In the case of an interdependent self-construal, self-integrity depends significantly on a person’s efficiency in meeting standards, rules, and expectations in a given situation or social role. Inconsistency is not uncomfortable for such a type and does not induce distress (Cross et al. 2003). On the contrary, it is the ability to flexibly modify one’s behavior according to the requirements of the current situation that is a sign of maturity for such individuals (Cross et al. 2003; Markus and Kitayama 1991). The true self is derived in this case from close relationships and thus remains context sensitive or even elusive (Suh 2007; Markus and Kitayama 1991). In addition, people with an interdependent self-construal may manifest greater diversity and complexity of self-representations as a consequence of incorporating attributes of close others within their own self (Aron et al. 1995; Church et al. 2008).

Another manifestation of the independent–interdependent differentiation may be found in accessibility of identity content. Individuals with an independent self-construal have a deep awareness of their own abilities and attributes (Markus and Kitayama 1991), and a highly salient self-schema (Głuchowska 2005). One would therefore expect them to have a heightened accessibility of self-representations, including those that constitute their identity. A very similar conclusion is suggested by the findings of Kozielecki (1986), who noted that knowledge articulation is largely based on cognitive differentiation. The findings of Witkin et al. (1954, 1962 as cited in Schenkel 1975) also link high awareness of one’s needs, feelings, and attributes to field independence. Both cognitive differentiation and field independence have been shown to be related to an independent self-construal (see Hannover et al. 2006; Kühnen et al. 2001; Kühnen and Oyserman 2002; Stapel and Koomen 2001; Woike and Matic 2004).

In the case of an interdependent self-construal, information about one’s own traits and abilities is derived from cues that are present in a particular social context (e.g., others’ approval) (Markus and Kitayama 1991). Also, clarity of knowledge about others exceeds clarity of self-knowledge (Głuchowska 2005). Therefore, we may expect those with an interdependent self to show reduced accessibility of identity content. Some support for the above contention is provided by the study of Gabriel et al. (2007), who demonstrated that people with an independent self-construal experienced greater self-confidence (i.e., feeling like they know themselves) than those with an interdependent self-construal. However, the self-confidence of the latter increased when close others were salient.

More complicated is the relationship between self-construal and valuation of identity content. Empirical evidence has shown that self-esteem is positively correlated with an independent self-construal, whereas an interdependent self-construal is related to lower self-esteem (e.g., Lam 2005; Morisaki 1997; Singelis et al. 1999). Such conclusions, however, need to be viewed with caution. It turns out that differences in self-esteem as a function of self-construal become apparent only in explicit self-esteem, but not in implicit self-esteem (Hannover et al. 2006; Kitayama and Uchida 2003). Moreover, given the controversy about the universality of self-enhancement, it could also be argued that the reported self-esteem differences may be influenced by the research focus and measurement tools (e.g., Heine et al. 1999; Heine 2005; Sedikides et al. 2003, 2005).

### Summary and Hypotheses

Theoretical and empirical reports on the possible relationships between self-construal and personal identity described above seem to be particularly striking if we consider the normative idea prevalent in the identity literature. A well-established identity, according to many theorists and researchers, is based on the perception and experience of one’s own distinctiveness, separateness, coherence, and continuity (e.g., Erikson 1963; Marcia 1966; Oleś 2008; Schachter 2002; Sokolik 1996; Vignoles et al. 2006). Since stability, coherence, specificity, separateness, and self-worth are some of the basic motives underlying personal identity formation, one would expect that they are ubiquitous, regardless of individual characteristics or socio-cultural context. However, it appears that they do not always play an equally important role, and that self-construal may be the factor that modifies their adaptive significance. Such a conclusion is suggested, for example, by the research of Cross et al. (2003), who analyzed the importance of cross-situational self-consistency for life satisfaction in people showing varying degrees of interdependence with others. The researchers pointed out that an interdependent self-construal is associated with lower coherence of self-view and increased tolerance to its inconsistency. In the case of an independent self-construal, maintaining coherence is a necessary condition for confirming one’s own self, and for this reason it is an index of maturity, integration, and unity of self, and is associated with well-being. Also encouraging are the findings by Kwan et al. (1997) and Okazaki (2002) that challenge the importance of self-esteem in the well-being of people with an interdependent self-construal. Although self-esteem reflects an aspect of well-being of those with an interdependent self, its importance is subordinate to a sense of connectedness, mutual understanding, and social acceptance (e.g., Lun et al. 2008; Suh et al. 2008; Uchida et al. 2004). Hence, a decrease in self-esteem is less damaging to the well-being of individuals with an interdependent self-construal than for those with an independent self-construal (Kwan et al. 1997; Okazaki 2002; see also Brockner and Chen 1996; Nakashima et al. 2008). In line with the cited studies are widely held assumptions that for people with an independent self-construal their own self-evaluations are essential and matter more than others’ evaluations of them. Therefore, we may expect such people to be especially motivated to maintain, validate, and enhance their self-evaluations (Kitayama and Imada 2008).

Despite the importance of the above studies and their contributions, we are still far from a model that integrates distinct self-construals and structural features of identity as contributors to well being. This paper is aimed to fill this gap. The above explorations may be summarized and concluded with the following conjecture: The adaptive value (i.e., consequences for subjective well-being) of various structural features of identity depend on the compatibility between an individual’s level of identity development and motives congruent with his or her dominant self-construal (see also Miluska 2006). People are motivated to develop and maintain an identity that provides them with a sense of continuity, coherence, boundaries, uniqueness, and self-worth. However, the optimal level of satisfaction of these senses will vary depending on how the self is construed: as socially embedded or as an independent entity.

Taking as a starting point the above premises, two hypotheses have been formulated. First, it was expected that the relationship between features of identity structure and subjective well-being in people with an independent self-construal would be stronger than in those with an interdependent self-construal. Second, it was expected that self-construal would act as a mediator between structural features of identity and subjective well-being.

## Method

### Participants

The sample included 226 Polish university students in different faculties of study (55.8 % female and 44.2 % male), whose age ranged from 18 to 28 years (*M* = 21.01, *SD* = 2.30).

### Measures

#### Self-Construal

Self-construal was assessed via an adapted version of the Self-Construal Scale (SCS; Singelis 1994). The Singelis’ scale is a 24-item measure, with two 12-item subscales each assessing independent and interdependent self-construal. The Polish modified version of the SCS was developed through translation and back translation process (Pilarska 2011)*.* In its final version, the scale includes 18 items (9 items in each subscale) evaluated on a seven-point scale, ranging from *strongly disagree* to *strongly agree*. Cronbach’s alpha reliability coefficients for the two subscales were *α* = 0.70 for independent self-construal and *α* = 0.72 for interdependent self-construal, which is comparable to previous reports on the original version of the SCS (*α* = 0.70 and *α* = 0.74 for the independent and interdependent subscales, respectively; Singelis 1994).

#### Identity Structure

To measure dimensions of identity structure the Multidimensional Questionnaire of Identity (MQI) (Pilarska 2012) was employed. The questionnaire consists of six subscales measuring the degree of accessibility, specificity, separateness, coherence, stability, and valuation of identity content, including a total of 38 items. All items are evaluated on a four-level scale ranging from *strongly disagree/never* to *strongly agree/always*. Test items were formulated after an extensive review of the literature and then selected by judges to match the theoretically important properties of the investigated construct. Next, items were empirically examined through a series of statistical analyses. A confirmatory factor analysis was conducted, designed to verify the six-factor structure of the MQI. The analysis was performed in a pilot study involving 411 subjects (*M* = 21.10 years, *SD* = 2.33 years). The chi-square test turned out to be significant, *χ* ^2^(650) = 1318.39, *p* < 0.01. However, it must be remembered that the chi-square test is highly dependent on such factors as, e.g., sample size, number of variables, number of free parameters, and deviations from the normal distribution of the data, which may be the reason for less satisfactory results. Therefore, it is prudent to examine other measures of fit (Zakrzewska 2004). Other fit indices for this model were acceptable (*χ* ^2^/*df* = 2.03, GFI = 0.85, AGFI = 0.83, RMSEA = 0.05, RMR = 0.04). The Cronbach’s alpha reliability coefficients for individual subscales were as follows: accessibility, *α* = 0.79; specificity, *α* = 0.79; separateness, *α* = 0.66; coherence, *α* = 0.86, stability, *α* = 0.63; valuation, *α* = 0.74. Table 2 presents sample items from particular subscales.

**Table 2:** Sample items from the Multidimensional Questionnaire of Identity

Subscale	Sample items
Accessibility	I know exactly what I feel and what I want
	I feel like I am losing contact with myself
Specificity	I prefer situations where I can mark my individuality to situations where I disappear in the group
	I feel that I am not distinguished by anything in particular from other people
Separateness	I easily share and actually experience the feelings and sentiments of others
	It happens that I perceive my close one as an important part of my self
Coherence	I have a feeling of inner harmony and order
	I feel like I am falling into pieces
Stability	I know that time passes and I am changing, but still I am sure that I am the same person as before
	I feel that I was once a very different person than I am now
Valuation	I like myself regardless of my shortcomings
	It happens that I am giving up the fight for something, as not to risk a failure

#### Positive and Negative Affect

The affective components of subjective well-being were measured with the positive and negative affect subscales (10 items each) taken from the Positive and Negative Affect Schedule – Expanded Form (PANAS-X) by Watson and Clark (1994). The participants were asked to indicate on a five-point scale, ranging from *very slightly or not at all* to *extremely*, the extent to which they, in general, experienced various emotional states. Reliability of the scales ranges from *α* = 0.83 to *α* = 0.90 for the PA scale and from *α* = 0.85 to *α* = 0.90 for the NA scale. The test–retest reliability coefficients fall in the range from *r* = 0.43 to *r* = 0.70 for PA and from *r* = 0.41 to *r* = 0.71 for NA (Watson and Clark 1994). Psychometric properties of the Polish version of PANAS-X are comparable to those of the original version: the reliability and stability indices are *α* = 0.86 and *r* = 0.53 for the PA scale, and *α* = 0.90 and *r* = 0.62 for the NA scale (Fajkowska and Marszał-Wiśniewska 2009). In the present study, the reliability coefficients for the PA scale was *α* = 0.77 and *α* = 0.90 for the NA scale.

#### Satisfaction with Life

Satisfaction with life is the cognitive component of subjective well-being and refers to the global assessment of the quality of one’s life based on one’s own criteria (Diener et al. 1985). The Satisfaction with Life Scale (SWLS) by Diener et al. (1985) contains five statements defining the overall degree of satisfaction with one’s past life. Every item is presented on a seven-point scale, allowing for a range of responses from *strongly disagree* to *strongly agree*. The scale’s reliability, as measured by the Cronbach’s alpha, is 0.87 and the reliability of the test–retest is *r* = 0.82 (Diener et al. 1985). The reliability index for the Polish version of the SWLS is *α* = 0.81 and the stability index is *r* = 0.86 (Juczyński 2001). In the present study, the reliability coefficient for this scale was *α* = 0.76.

### Procedure

The study was conducted in a collaborative mode, ensuring anonymity and confidentiality. The subjects were informed about the purpose of the study. The consent of each individual was the condition of participation in the research. All participants received identical instructions and filled out, in a consecutive order, the Self-Construal Scale, the Multidimensional Questionnaire of Identity, the Positive and Negative Affect Schedule, and the Satisfaction with Life Scale.

## Results

### Gender Differences

The analysis of Student’s *t* test and Mann–Whitney *U* test revealed that men scored higher than women in terms of accessibility (*M*_men_ = 11.97 [*SD* = 2.75] vs. *M*_women_ = 11.44 [*SD* = 2.17]), *U* = 5087.50, *p* < 0.05, *g*_*r*_ = 0.19; specificity (*M*_men_ = 13.90 [*SD* = 3.77] vs. *M*_women_ = 12.38 [*SD* = 3.16]), *t*(224) = 3.29, *p* < 0.001, *d* = 0.44; separateness (*M*_men_ = 11.37 [*SD* = 3.05] vs. *M*_women_ = 10.27 [*SD* = 3.13]), *t*(224) = 2.65, *p* < 0.01, *d* = 0.35; and valuation (*M*_men_ = 12.98 [*SD* = 2.88] vs. *M*_women_ = 11.54 [*SD* = 2.72]), *t*(224) = 3.85, *p* < 0.001, *d* = 0.51. Moreover, women scored significantly higher than men on the negative affect scale (*M*_women_ = 27.72 [*SD* = 8.27] vs. *M*_men_ = 24.47 [*SD* = 7.33]), *t*(224) = 3.09, *p* < 0.01, *d* = 0.41. In terms of other variables, no significant gender differences were observed.

### Zero-Order Correlations

Table 3 presents intercorrelations for all variables. The obtained results are generally in line with expectations. Independent self-construal correlated positively with all features of identity structure. The strongest positive relation was observed between independent self and specificity (*r* = 0.45, *p* < 0.001); other links were weak and moderate. Interdependent self-construal was negatively correlated with specificity, separateness, and valuation of identity content. The strongest negative link was found between interdependent self and separateness (*r* = −0.43, *p* < 0.001). Moreover, all dimensions of identity structure correlated negatively with negative affect. Accessibility, specificity, separateness, coherence, and valuation were positively correlated with positive affect. Finally, accessibility, specificity, coherence, stability, and valuation were positively correlated with satisfaction with life. The strongest relationship occurred between valuation and satisfaction with life (*r* = 0.51, *p* < 0.001). Other links were mostly of moderate strength. These results are a starting point for more detailed analyses of the adaptive value of identity structure in the context of its interaction with self-construal.

**Table 3:** Zero-order correlation matrix, means, and standard deviations for all variables

Variable	1	2	3	4	5	6	7	8	9	10	11
1. IND	–										
2. INT	−0.10	–									
3. ACC	0.22***	−0.02	–								
4. SPE	0.45***	−0.34***	0.19**	–							
5. SEP	0.15*	−0.43***	0.16*	0.27***	–						
6. COH	0.23***	−0.09	0.80***	0.25***	0.30**	–					
7. STA	0.19**	0.02	0.39***	0.08	0.10	0.37***	–				
8. VAL	0.36***	−0.18**	0.48***	0.56***	0.33***	0.56***	0.23***	–			
9. NA	−0.17*	0.02	−0.40***	−0.20**	−0.30***	−0.44***	−0.22***	−0.50***	–		
10. PA	0.47***	−0.12	0.36***	0.48***	0.13*	0.33***	0.13	0.48***	−0.05	–	
11. SWL	0.18**	0.01	0.37***	0.31***	0.09	0.48***	0.23***	0.51***	−0.36***	0.34***	–
*M*	43.85	41.10	11.68	13.06	10.76	20.97	5.08	12.18	26.28	35.50	21.44
*SD*	6.72	6.61	2.45	3.52	3.14	4.76	1.91	2.88	8.02	5.26	5.27

*N* = 226

*IND* independent self-construal; *INT* interdependent self-construal; *ACC* accessibility; *SPE* specificity; *SEP* separateness; *COH* coherence; *STA* stability; *VAL* valuation; *NA* negative affect; *PA* positive affect; *SWL* satisfaction with life

*** *p* < 0.001, two-tailed. ** *p* < 0.01, two-tailed. * *p* < 0.05, two-tailed

### Relationships Between Identity Structure and Well-Being in the Context of Independent and Interdependent Self-Construal

It was assumed that the dominance of an independent self-construal would be linked to stronger associations between the structural features of identity and the components of subjective well-being. On the other hand, in people with an interdependent self-construal, these relationships were expected to be weaker.

To study the correlated variables, Pearson’s linear correlation coefficient was used. The resulting correlation coefficients are shown in Table 4. In order to verify whether the groups distinguished by the predominant self-construal differed significantly with respect to the strength of the relationship between various structural features of identity and the components of well-being, Fisher’s Z-test was used. The compared groups were composed of subjects who obtained extremely low scores on one of the types of self-construal, and yet scored extremely high on the other type of self-construal. Probable-error criterion was used (0.6745 × *SD)* as a cut-off point. The high results were equal to or greater than the sum of the mean score and the probable error, and the low results were lower than the difference between the mean score and probable error, or equal to this difference.

**Table 4:** Correlation matrix between identity structure and well-being among independents and interdependents

Variable	1	2	3	4	5	6	7	8	9
1. ACC	–	0.22	0.42	0.80***	0.48*	0.55**	−0.34	0.27	0.43*
2. SPE	0.57**	–	0.16	0.29	−0.05	0.40	−0.22	0.30	0.19
3. SEP	−0.02	0.21	–	0.57**	0.02	0.42	−0.40	−0.12	0.19
4. COH	0.73***	0.68***	−0.01	–	0.36	0.46*	−0.25	0.20	0.24
5. STA	0.55*	0.30	−0.04	0.38	–	0.05	−0.41	−0.24	−0.08
6. VAL	0.67***	0.73***	0.15	0.81***	0.36	–	−0.54**	0.15	0.40
7. NA	−0.45*	−0.55*	−0.39	−0.52*	0.06	−0.62**	–	0.42	−0.09
8. PA	0.61**	0.44	−0.08	0.63**	0.59**	0.68***	−0.28	–	0.52*
9. SWL	0.46*	0.50*	−0.20	0.72***	0.31	0.61**	−0.42	0.44	–

Intercorrelations for independents sample (*n* = 20) are presented below the diagonal, and intercorrelations for interdependents sample (*n* = 22) are presented above the diagonal

*ACC* accessibility; *SPE* specificity; *SEP* separateness; *COH* coherence; *STA* stability; *VAL* valuation; *NA* negative affect; *PA* positive affect; *SWL* satisfaction with life

*** *p* < 0.001, two-tailed. ** *p* < 0.01, two-tailed. * *p* < 0.05, two-tailed

The conducted analyses show that the relationships between the features of identity structure and the well-being components differ for individuals with distinct self-construals. There were strong positive associations of coherence with satisfaction with life (*r* = 0.72, *p* < 0.001) and with positive affect (*r* = 0.63, *p* < 0.001), as well as a strong negative correlation between coherence and negative affect (*r* = −0.52, *p* < 0.001) present in the group of people with a dominant independent self-construal. No such links were observed in individuals with a predominantly interdependent self-construal (*p* > 0.05). Tests of the significance of the difference between correlation coefficients obtained for both groups showed that the relation between identity coherence and life satisfaction was significantly stronger among individuals with a predominantly independent self, *z* = 1.99, *p* < 0.05, *q* = 0.66. Strong positive correlations appeared also between valuation and both positive affect (*r* = 0.68, *p* < 0.01) and life satisfaction (*r* = 0.61, *p* < 0.01), and between stability and positive affect (*r* = 0.59, *p* < 0.001), but again, only among those with a predominantly independent self. Tests of the significance of the difference between correlation coefficients in the two groups indicated that the relationship between valuation and positive affect, as well as the relation between stability and positive affect, was significantly stronger among individuals with a predominantly independent self, *z* = 2.03, *p* < 0.05, *q* = 0.68, and *z* = 2.77, *p* < 0.01, *q* = 0.93, respectively. In individuals with a predominantly independent self, strong correlations were also observed between accessibility of identity content and both positive affect (positive correlation, *r* = 0.61, *p* < 0.01) and negative affect (negative correlation, *r* = −0.45, *p* < 0.05). These correlations did not occur in individuals with a predominantly interdependent self (*p* > 0.05). Moreover, in the group with a predominantly independent self, specificity was strongly negatively associated with negative affect (*r* = −0.55, *p* < 0.05) and strongly positively associated with satisfaction with life (*r* = 0.50, *p* < 0.05). In the group with a predominantly interdependent self, the correlations between specificity and the well-being components did not reach statistical significance (*p* > 0.05).

In both groups, valuation was significantly negatively correlated with negative affect (*r* = −0.62 and *r* = −0.54, *p* < 0.01), and accessibility was significantly positively correlated with life satisfaction (*r* = 0.46 and *r* = 0.43, *p* < 0.05). Correlations between the remaining variables did not reach the level of statistical significance in any of the analyzed groups.

### Path Analysis

To verify the hypothesis of the mediating role of self-construal in the relationship between the features of identity structure and well-being, a path analysis was performed.

**Fig. 1:**
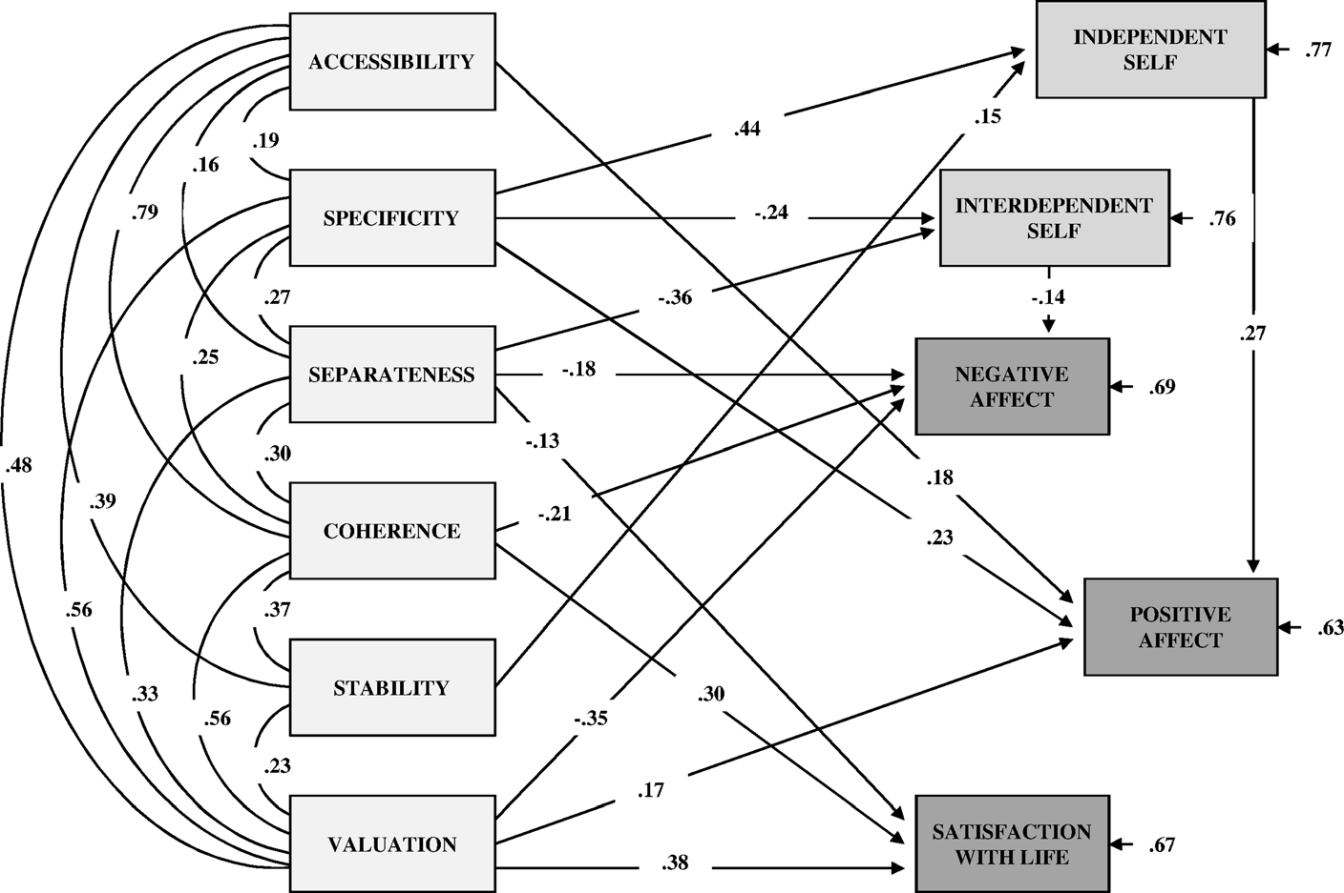
The final model. *N* = 226. Standardized path coefficients are shown; all these coefficients were significant at *p* < 0.05 or less

The path model depicted in Fig. 1 showed acceptable fit level as judged by goodness of fit estimates. The result of the chi-square test, *χ* ^2^(27) = 41.99, *p* < 0.05, was statistically significant, but—again referring to remarks by Zakrzewska (2004)—one should take into account the fact that this result is highly dependent on sample size, number of variables, number of free parameters, and deviations from the normal distribution of observable variables. The chi-square to df ratio of 1.56 indicated a very good fit of the model. The GFI and AGFI were both above their desired levels (GFI = 0.97 and AGFI = 0.92). The results for RMSEA and RMR fell within range of acceptable values (RMSEA = 0.05 and RMR = 0.045). The upper confidence limit for RMSEA was less than the value of 0.08, and the RMSEA-based test of close fit also indicated good fit (*p* = 0.46). Overall, the various goodness-of-fit statistics indicate that the model fits the empirical data well.

As shown in Fig. 1, almost all of the features of identity structure correlated significantly and positively with each other. Two exceptions were the lack of significant correlation between specificity and stability, and between stability and valuation of identity content. The strongest link was observed between accessibility and coherence (*r* = 0.79, *p* < 0.05), and the weakest was between accessibility and separateness (*r* = 0.16, *p* < 0.05). Other links were of moderate or weak strength.

Within the endogenous variables, significant regression coefficients were found for (a) independent self and positive affect (*β* = 0.27), and (b) interdependent self and negative affect (*β* = −0.14). Significantly and positively related to independent self were two of the six analyzed dimensions of identity structure: specificity (*β* = 0.44) and stability (*β* = 0.15). The unique variance for independent self was 77 %. Significantly but negatively associated with interdependent self were also two of the six analyzed structural features of identity: specificity (*β* = −0.24) and separateness (*β* = −0.36). The unique variance for interdependent self was 76 %.

Accessibility had a direct effect on positive affect (*β* = 0.18). Coherence had a direct effect on negative affect (*β* = −0.21) and satisfaction with life (*β* = 0.30). Valuation had a direct effect on negative affect (*β* = −0.35), positive affect (*β* = 0.17), and satisfaction with life (*β* = 0.38). Accessibility, coherence, and valuation only directly affected the well-being components, so the indicated path values were estimates of the total effects. Stability was related only to positive affect, but this effect was mediated through independent self. The size of this effect was 0.04 (*p* < 0.05). Specificity had both direct (*β* = 0.23) and indirect effects on positive affect, operating indirectly via independent self. The size of the indirect effect was 0.12 (*p* < 0.001). The total effect of specificity on positive affect was therefore 0.35. Specificity indirectly (via interdependent self) affected also negative affect. The size of this effect was 0.03 (*p* < 0.06), and was the estimate of the total effect of specificity on negative affect.

Separateness had direct effects on negative affect (*β* = −0.18) and life satisfaction (*β* = −0.13). Separateness did not affect satisfaction with life through any other variable, and therefore the value of −0.13 remained the total effect of separateness on life satisfaction. Negative affect was influenced by separateness also indirectly through interdependent self. The size of this effect was 0.05 (*p* < 0.05), so the total effect of separateness on negative affect was −0.13 (suppression effect).

As a whole, the model explained 31 % of variance in negative affect, 33 % of variance in life satisfaction, and 37 % of variance in positive affect.

## Discussion

The aim of this study was to investigate the adaptive value of structural features of identity, described in terms of subjective well-being, in the context of independent and interdependent self-construal.^3^

As expected, the results demonstrated that there were differences between independent and interdependent individuals in how the structural features of identity impacted on affective and cognitive components of subjective well-being. Correlation analyses revealed that in subjects with a predominantly independent self-construal, increases in accessibility, specificity, coherence, and valuation of identity content were associated with a decrease in negative affect, an increase in positive affect, and an increase in experienced life satisfaction. Moreover, a growth of a sense of continuity was accompanied in this group by an increase in positive affect. Among individuals with a predominantly interdependent self-construal, significant links (consistent with those described above with respect to direction) were found only between accessibility of identity content and satisfaction with life, and between valuation of identity content and negative affect intensity. Assessments of significance of the difference between correlation coefficients in both groups indicated that the correlation between life satisfaction and coherence of identity content, and the correlations of stability and of valuation of identity content with the intensity of positive emotional experiences were significantly stronger among individuals manifesting a predominantly independent self. The obtained results are consistent with findings of Cross et al. (2003) and Gore and Cross (2010), showing that the significance of cross-situational self-consistency and compatibility among one’s goals (goal coherence) for life satisfaction is a function of the degree of interdependence of self-construal (cf. Locke and Christensen 2007). It turns out that these findings apply to the coherence of identity as well: In the case of individuals with an independent self-construal, a sense of identity coherence had beneficial effect on all components of well-being, but in individuals with an interdependent self such links were not observed. Corresponding results at the cultural level were obtained by Suh (2002), whose study showed that among Korean students, compared to United States students, subjective well-being is less predictable from levels of identity consistency. The reports of Kwan et al. (1997) and Okazaki (2002), suggesting a reduction of the importance of self-esteem for well-being in people with an interdependent self-construal, also correspond to the results obtained in this study. Although self-worth was strongly associated with emotional well-being and life satisfaction in people with an independent self-construal, in the case of interdependent individuals its role was limited to the relationship with negative affect. It would seem that, regardless of self-construal, people with high self-worth experience less negative affect than low self-worth individuals. Yet, it appears that high self-worth promotes positive affect and life satisfaction only for those with an independent self-construal. These results could explain why individuals with an independent self-construal have a stronger tendency to defend and strive for self-esteem, and support the notion that independent individuals self-enhance more than interdependent ones (e.g., Heine et al. 1999; Suh 2000).

The model describing the mediating effects of independent and interdependent self-construals on the relationship between identity structure and subjective well-being was also verified by means of path analysis. The obtained results allow the conclusion that the features of identity structure influence the affective and cognitive components of well-being in a direct way, as well as through self-construal. A sense of uniqueness and separateness of identity content had both direct and indirect effects on subjective well-being. The impact of accessibility, coherence, and valuation of identity content on subjective well-being was exclusively direct, whereas a sense of continuity affected subjective well-being only indirectly. The effects mediated by independent and/or interdependent self-construal that are of particular interest here, show that:

- The higher the level of specificity of identity content, the higher the intensity of positive affect, but this effect is stronger at higher levels of independent self-construal.
- When the self is highly interdependent, an increase in specificity of identity content is predictive of negative affect growth.
- The higher the level of separateness of identity content, the lower the intensity of negative affect, but this effect is weaker at higher levels of interdependent self.
- When the self is highly independent, an increase in stability of identity content is predictive of positive affect growth.

The research results described above show that the role of self-construal becomes evident in regard to the consequences of specificity, separateness, and stability of identity content, when confronted with the affective components of well-being. Moreover, in the case of associations between (a) stability and positive affect, and (b) specificity and negative affect, total mediations were observed.

The adaptive importance of a sense of continuity over time, emphasized in literature (Brzezińska and Kofta 1974; Chen et al. 2007; Grzegolowska-Klarkowska and Zolnierczyk 1988), in the light of the present study, appears to be entirely a derivative of the relationship between independent self-construal and stability of identity content. This observation is even more important since stability did not directly affect any other component of well-being—the mediated effect turned out to be the only path between a sense of continuity and well-being. This result is in line with Church et al.’s (2006) findings that individualistic cultures, as compared to collectivistic cultures, have stronger implicit beliefs regarding the traitedness of behavior (i.e., consistency of behavior over time). It also corresponds to the observation by Campbell et al. (1996) that, among Canadians, self-concept clarity is more closely associated with self-esteem, than it is for Japanese.

In the case of specificity of identity content, an effect mediated by interdependent self-construal was revealed. The weakening of a sense of one’s uniqueness was predictive of the interdependence of self-construal, which, in turn, prevented the intensification of negative affect. However, it is worth noting that specificity affected also the positive affect component of subjective well-being, both directly and indirectly through independent self-construal. Once aggregated, these effects indicated that a strong sense of uniqueness was the major single determinant of a high level of positive affect, and this influence was stronger than it was with respect to negative affect. Thus, one may conclude that for the emotional well-being of the study participants, it was still more favorable to experience oneself as a unique individual. These findings provide considerable support for the distinctiveness principle, which is postulated to be a universal human need (e.g., Breakwell 1993; Vignoles et al. 2000). At the same time, they offer some insight into individual differences in the relative strength of the motivational pressure towards distinctiveness. The present study reveals that distinctiveness is less beneficial to emotional well-being among people with an interdependent self-construal. Among such individuals, feeling highly unique is associated with an increase in negative emotions, most likely because it frustrates a sense of belongingness and similarity to others (see Vignoles 2000; Vignoles et al. 2000).

With regard to a sense of one’s own boundaries, the situation seems similar. The weakening of a sense of separateness led to increased levels of negative affect, although this effect turned out to be suppressed to some extent by interdependent self-construal. These results are consistent with the notion that, although the functional role of separateness remains universal (e.g., Chirkov 2007; Skowron et al. 2003), individuals with an interdependent self-construal have less rigid self-other boundaries and are less sensitive to boundary threat (see Cross et al. 2000; Wong 2010). In addition, separateness was negatively predictive of experienced life satisfaction. Comparison of the two described paths leads to the conclusion that a strong sense of separateness diminishes the intensity of experienced negative emotional states to the same extent to which it is detrimental for life satisfaction.^4^

## Conclusion

The analysis of the obtained results confirms that reliance on the findings of self-construal theory as means for determining identity-related criteria of emotional well-being and life satisfaction is well founded. The comparison of the correlations between structural features of identity and components of well-being for individuals with a dominant independent self-construal and those with a dominant interdependent self-construal suggested that a weakening of these correlations may be considered characteristic of interdependent individuals. The results of path analysis further revealed the mediating role of self-construal. While these new observations are not contradictory to the well-established view that a sense of self-worth, uniqueness, boundaries, coherence, continuity, and a sense of having inner content are beneficial for subjective well-being, it nevertheless holds true that this general view requires some qualifications. It is important to recognize that independent self-construal reinforces the beneficial effect of a unique and stable identity, whereas interdependent self-construal reduces the harmful effects of the weakening of a sense of uniqueness and separateness. These results deserve special emphasis. For although much effort in the literature is devoted to describing identity-related determinants of effective psychosocial adaptation, relatively little research attention is dedicated to potential moderators of the impact of identity structure on adjustment outcomes. Still, growing up in an environment that is either individuation-oriented or affiliation-oriented impinges on the content of socio-cultural transmission and ultimately determines the way individuals conceive of themselves. Since identity formation involves identification with certain values, goals, and beliefs, it may be assumed that self-construal, as a medium for the social world, affects the motivational principles of personal identity. Construing one’s own self as independent or interdependent can be expected to produce a certain kind of motivational tension in the area of identity formation and shape the nature of identity crises. In this context, an individual’s level of well-being may be considered as contingent on one’s success in both achieving goals pertaining to one’s dominant self-construal and satisfying one’s identity-related needs.

## Footnotes

1 Some other authors go as far as to take the opposite position, suggesting instead that various identities make up one’s self-concept (e.g., Oyserman et al. 2012). In their conceptualization, the term identity is used as equivalent to traits, roles, and social relations.

2 In one of their works, Markus and Kitayama (2003) emphasize that the differences between independent and interdependent self-construal refer to various “theories of being” and “theories of reality”. Self-construals can therefore be regarded as transmitters of a specific message on what is important in the world and what in this world means “to be”.

3 Although it was not the main focus of the current research, it is worth noting that the obtained results concerning the relationship between identity structure and well-being favor the conceptualization of personal identity derived from the classical writings of Erikson (1963, 1968) and his followers (e.g., Blasi and Glodis 1995; Marcia 1966). As such they challenge postmodern suggestions about the illusion (or the absence) of a unified, coherent, and stable identity, as well as the statements that personal sameness and continuity should no longer be considered the distinguishing mark of maturity and well-being (e.g., Gergen 1991; Giddens 1991; Hermans 2003; see also Schachter 2004).

4 These divergent outcomes of separateness may imply that people do not always consult their emotions when evaluating life satisfaction and that affective cues are not sufficient for making the satisfaction judgment (see Suh et al. 2008).
